# Water, Sanitation, and Hygiene: Linkages with Stunting in Rural Ethiopia

**DOI:** 10.3390/ijerph16203793

**Published:** 2019-10-09

**Authors:** Corina Shika Kwami, Samuel Godfrey, Hippolyte Gavilan, Monica Lakhanpaul, Priti Parikh

**Affiliations:** 1 Department of Civil, Environment and Geomatic Engineering, University College London, Chadwick Building, London WC1E6BT, UK; c.kwami@ucl.ac.uk (C.S.K.); h.a.gavilan@gmail.com (H.G.); 2 United Nations Children’s Fund (UNICEF), Regional Water and Sanitation Advisor for East and Southern Africa, Nairobi 00100, Kenya; sgodfrey@unicef.org; 3 UCL-Great Ormond Street Institute of Child Health, University College London, London WC1N 1EH, UK; m.lakhanpaul@ucl.ac.uk; 4 Whittington Health NHS Trust, London N19 5NF, UK

**Keywords:** stunting, WASH, child health, hand-washing, environmental health, clean water, evidence-based policy-making, behaviour change, undernutrition

## Abstract

Stunting is a global burden affecting nearly 160 million children younger than five years of age. Whilst the linkages between nutrition and stunting are well recognized, there is a need to explore environmental factors such as water and sanitation, which may influence feeding practices and result in potential infection pathways. This paper explores the linkages between stunting and water, sanitation and hygiene (WASH) factors in Ethiopia, which is a relatively understudied context. The research draws upon baseline data for children under the age of five from 3200 households across four regions in Ethiopia as part of a wider study and integrated program led by the United Nations Children’s Fund (UNICEF). Using World Health Organization (WHO) z-scoring, the average stunting rate in the sample is 47.5%. This paper also takes into account demographic and social behavioural factors such as the age, gender of children, and gender of the primary caregiver, in addition to handwashing behaviour and drinking water facilities. The evidence recommends efforts to improve handwashing behaviour for mothers and children with a focus on access to clean water. Higher stunting rates with an increase in the age of children highlight the need for continued interventions, as efforts to improve nutrition and WASH behaviours are most effective early on in promoting long-term health outcomes for children.

## 1. Introduction

In 2016, 158 million children younger than 5 years were mildly, moderately, or severely stunted [1]. Stunted growth reflects the failure of reaching average linear growth potential as a result of suboptimal health, nutritional, and environmental conditions [2]. Overall, the cost of malnutrition is estimated to range from 2% to 16% of gross domestic product (GDP) in the most affected countries, which has implications for sustainability and efforts to reduce poverty [3]. Sustainable Development Goal 2.2 has already made undernutrition a priority, as both a barrier to achieving sustainable development and as an indicator of progress in development [4]. Undernutrition is an underlying cause of 3.1 million child deaths annually [5]. Therefore, improving nutrition is essential to fight poverty, and thereby contributes to efforts to reduce inequality and improve sustainability capabilities. 

Globally, stunting rates are declining slowly with the largest improvements in Asia and Latin America. However, Africa is the only region where the number of stunted children has risen from 50% to 59% between 2000–2016, which highlights the urgency of the challenge [6]. In Ethiopia, stunting rates as high as 40.4% were reported in 2015 [7], with 28% of all child mortality associated with undernutrition [8]. The annual costs associated with child malnutrition are estimated to amount up to 16.5% of Ethiopia’s GDP [8]. With 44% of health costs associated with undernutrition for children under one in Ethiopia, addressing enabling conditions and structural determinants presents an opportunity to improve health and reduce overall health costs in a country that has a disproportionately high burden of stunting [8]. 

The definition of nutrition defined by the United Nations Children’s Fund (UNICEF) conceptual framework provides a multifactorial understanding of nutrition in which to frame stunting: “Nutritional status is influenced by three broad factors: food, health and care [9]. Optimal nutritional status results when children have access to affordable, diverse, nutrient-rich food; appropriate maternal and child-care practices; adequate health services; and a healthy environment including safe water, sanitation and good hygiene practices” [9]. The World Health Organization (WHO) defines stunting as “the impaired growth and development that children experience from poor nutrition, repeated infection, and inadequate psychosocial stimulation” [10]. This definition does not account for genetic variation. Children are defined as stunted if their height-for-age is more than two standard deviations below the WHO Child Growth Standards median. There has been a call for a reframing of undernutrition beyond the provision of adequate food (quantity) and improving its quality (diversity) in order to have a better balance of understanding, which may influence the design of interventions [11]. The argument for focus on social and environmental factors is also supported by success stories in the reduction of stunting, as in the case of Peru. After a long stagnation period, Peru dramatically reduced its national and departmental stunting prevalence through a combination of social determinants and crosscutting factors. For other countries trying to improve nutrition, stunting reduction may be helped by lessons learnt from the adoption of anti-poverty policies and the sustained implementation of equitable crosscutting interventions, with focus on the poorest areas [12].

There is some research that renders the linkage between stunting and Integrated Water, Sanitation and Hygiene (WASH) practices more nuanced in many low and middle income countries (LMICs) in Africa and Asia. One study identified a lack of correlation with stunting, attributing it to what is presently classified as enteric environmental dysfunction. Unsanitary conditions have been linked with stunting through multiple mechanisms and pathways such as repeated diarrhea, infection pathways, and environmental enteric dysfunction (EED) [13]. Humphreys identified this as a strong linkage, as opposed to diarrhea, arguing that “tropical enteropathy now (EED) is caused by fecal bacteria ingested in large quantities by young children living in conditions of poor sanitation and hygiene [14]”. Humphreys argued that the provision of toilets and promotion of handwashing after fecal contact could reduce or prevent tropical enteropathy and its adverse effects on growth. This finding provides the foundation for a case for why the primary causal pathway from poor sanitation and hygiene to undernutrition is tropical enteropathy, and not diarrhea [14]. For prevention, technologies and behaviours that serve to safely contain excreta such as preventing human contact and washing with soap at critical times (e.g., after defecation and before eating) is among recommendations to prevent increased stunting [15]. It has been argued through a systematic review of the literature that handwashing has a role to play in the reduction of diarrheal morbidity [16]. There is a long way to go, as only 19% of the population globally practice handwashing with soap, with a lack of clarity on the occasions in which handwashing is the most important—after defecation, before and after preparation of food and feeding, and for which family member—the mother, child, and other caregivers [13]. WASH infrastructure has the potential to provide access to clean water, which is essential to promote hand-washing practices. Since 2009, another review has explored the existing literature surrounding the proposed pathology and transmission of EED in infants, and confirms considerations for nutrition and WASH interventions to improve linear growth worldwide [17]. This is consistent with a cross-sectional study in India on household environment and stunted children that identified strong associations between WASH and stunting [18]. While there is significant research on stunting, the inclusion of WASH facilities and their correlation with stunting has been relatively understudied in the Ethiopian context.

The aim of this paper is to explore associations between stunting and WASH factors, accounting for descriptive factors (potential confounding factors identified in the literature) that are specific to the Ethiopian context in order to inform the design and implementation efforts that address stunting in context. Data for this project is baseline data as part of a wider five-year project jointly implemented by the Government of Ethiopia and UNICEF in collaboration with The Integrated Water, Sanitation and Hygiene (WASH), Multiple Use Water and Sanitation Services (MUS), and Community-Based Nutrition (CBN) program. The project focused on deploying an integrated approach to counter stunting that addresses nutritional, hygiene, and WASH factors. This paper begins with a summary of the literature on known determinants of stunting, showing where there is an opportunity to address the gap in the association with WASH in the Ethiopian context. The Methods and Materials section summarizes the approach from the context of the study within the wider integrated project and the approach used to explore WASH and other social determinants in the Ethiopian context using the baseline data collected by UNICEF. The Results section presents the correlations and results of linear regression, which are followed by a discussion that places the results in dialogue with the background literature, recommendations, and areas for further exploration. The paper concludes with a summary. 

### 1.1. Literature Review

Contrary to common belief, stunting is complex and not solely a result of food insecurity: many children in food-secure environments are stunted because of inappropriate feeding and care practices, poor health services, or and/or poor sanitation [5]. This section summarizes the demographic, nutritional, maternal health, and temporal factors to consider in efforts related to stunting. The section also presents the current gaps on research related to WASH determinants of stunting and frames the focus for the paper around investigating these gaps in the Ethiopian context.

Demographic factors, income growth, food production, and women’s education are important contextual considerations for long-term solutions to undernutrition [5]. Population density and the density of open defecation are factors that were identified to be strongly associated with stunting in a comparative analysis of 130 Demographic & Health Surveys (DHS) cross-sectional surveys [19]. There are several studies that report stunting as significantly less prevalent in families with higher socioeconomic status and increases with poverty, environmental factors, a non-utilization of health services, and lack of parental education [20,21,22,23]. Levels of parental education, and in particular maternal education, inversely affect stunting, as demonstrated through a cross-sectional analysis of the Demographic and Health Surveys for low and middle-income countries [24]. A study by the Ethiopian Health and Nutrition Research Institute highlights the need for nutrition education on appropriate feeding practices as a critical enabler of child nutrition [25]. The study also notes that the highest stunting rates are in regions in Ethiopia with surplus food, suggesting that the availability of food is not a critical factor in stunting, but that social factors may be stronger predictors for poor child growth. There is still a gap in the evidence base on the correlation between WASH factors and stunting in the Ethiopian context. This gap offers an opportunity to inform the design of future interventions that are grounded in an understanding of local needs and capacities.

While this study is focused on contextual factors, it would be remiss not to present the evidence related to stunting that considers the temporal dimension, namely that the prevalence of the most damaging effects of undernutrition occur during pregnancy and in the first two years of life [24]. This is consistent with the sixth report on the World Nutrition Situation [26], which calls for efforts to invest in maternal nutrition in order to improve behaviours associated with infant birth weight and child growth in the first two years of life [3]. The first 1000 days of an infant’s life are seen to be the most critical window [3]. The nutritional status of newborn infants shows strong linkages to the mother’s health and nutritional status, with an estimated half of child stunting first manifesting in utero [3]. Nonetheless, the increase in stunting rates commonly occurs between 0–2 years of age [3], and is related to a multitude of factors related to infant and young child feeding practices, such as non-exclusive breastfeeding and complementary feeding that is limited in quantity, quality, and variety, and other social, environmental factors [27]. In the Ethiopian context, an observational study has found that stunting develops during the weaning period until the maximum age of three, and then slightly declines thereafter [28]. 

In addition to nutritional factors, maternal health and temporal dimensions of stunting, demographic variables such as gender and socioeconomic status, as well as environmental factors are found to have links to stunting. A cross-sectional study in the northwest of Ethiopia links factors such as child age, occupational status of the household head, family size, and education level of the father with stunting [29]. Another study from the Amhara Region in Ethiopia notes that while stunting is more frequently found in children born to young mothers, stronger links are established with land ownership and water quality [30]. This study notes that female-headed households had a higher prevalence of malnutrition, and provides evidence for linkages between stunting and a shortage of farmland, limited livestock possessions, and a shortage of non-farm employment opportunities [30]. While economic factors are important to consider, there is evidence from a literature review on chronic malnutrition in Ethiopia that identifies an over-reliance on macroeconomic growth as a solitary factor toward undernutrition. Maternal education and WASH are among the second strongest factors statistically associated with stunting after optimal feeding recommendations, which were the most prominent predictors of stunting [31]. 

Other contextual factors such as ethnicity were found to predict chronic undernutrition, particularly when children are reared in disadvantaged ethnic groups. These results come from a cross-sectional comparative study in Nepal [32] as well as in Guatemala, where ethnicity was also found to correlate with stunting, particularly for children from ethnic groups that tended to have shorter stature [33]. There is also a likelihood that ethnicity could be highly varied with socioeconomic status and religion. Investigating these correlations and any collinearity would likely need to be supplemented with in-depth interviews and cultural anthropological methods to identify where and how these collinear mechanisms operate.

With regard to environmental factors such as access to water and sanitation, there is some evidence for an association with a reduction in the rate of diarrhea, which is also a predictor for child nutrition. One such study included a multi-village project in Ethiopia, where 11 villages were selected for interventions on health, education, WASH, or an integrated approach using health, education, and WASH [34]. The intervention group that receive integrated health, education, and WASH activities showed a significant reduction in stunting attributed to improvement in access to WASH services and maternal knowledge on causes of diarrhea and hygiene practices [34]. Hygiene practices can be linked to environmental cleanliness, the presence of actual toilets, and other environmental factors such as wetness and dryness (although the latter is beyond the scope of factors investigated in this review). This finding is consistent with a study of a population-based sample of women in the third trimester who were monitored until the infants were 12 months of age [35]. Open defecation, when linked to the absence of an actual toilet and/or insufficient use of actual toilets, was noted to be significantly associated with stunting in Ethiopia [36]. However, randomized controlled trials such as the large-scale Sanitation Hygiene Infant Nutrition Efficacy (SHINE) study identified weak or non-existent linkages between WASH and stunting [37]. Cumming and Curtis (2019) suggest the possibility that most trial participants already having access to basic latrines, improved drinking water sources, and showing low rates of open defecation at baseline may have resulted in weaker WASH and stunting linkages [38]. There is also a need for further investigation of specific WASH factors in relation to quality of access (access to facilities, handwashing activities) [39]. Taking into account these sources that provide evidence for the association between WASH and stunting, there is a lack of studies in the Ethiopian context that provide an assessment of WASH factors based on contextual, demographic factors and a relatively large sample on which to draw conclusions that can inform future interventions. The following sections will introduce an integrated approach to WASH that aims to address this research gap. 

### 1.2. Response to Stunting: Integrated Program

The integrated WASH, Multiple Use Water and Sanitation Services (MUS) and Community-Based Nutrition (CBN) program, a five-year project jointly implemented by the Government of Ethiopia and UNICEF, aims to respond to factors associated with stunting in an integrated manner. A key objective of this large-scale rural program is to address the combined risks of chronic malnutrition and poor access to WASH services and water for local agricultural production, which is linked to improved food security, child health, and reproductive and sexual health outcomes [1]. This five-year program (2012–2017) focused on community-based WASH interventions and the promotion of MUS with the aim of reaching 1.4 million people. The data for this analysis is drawn from the baseline survey data, and does not include end line measurements. This study presents the results of the investigation that examined the association of WASH factors in an analysis of stunting. The following Methods section introduces the terminology used, and describes the research plan, data collection, and analysis.

## 2. Materials and Methods

The analysis is a subset of a wider controlled trial that includes WASH and Multiple Use Services (MUS) activities integrated with Community-Based Nutrition (CBN) interventions, operational research, and the dissemination of knowledge products carried out by UNICEF and the Government of Ethiopia. Ethical clearance for the study was obtained by UNICEF from the Federal Ministry of Health and Federal Ministry of Water (August 2013). Both ministries also provided feedback and peer reviewed the questionnaire implemented in this study. This project is situated within the wider objective of increasing the impact on the reduction of stunting and other health outcomes through an integrated approach. Whilst water supply, sanitation, and hygiene interventions were implemented with a view to improving child health, it was anticipated that a significantly wider impact on stunting and food security would be secured by combining these interventions with a community-based nutrition (CBN) package. In the baseline stage, the objective of the wider project was to establish representative baseline data on the selected indicators in 30 WASH/CBN kebeles, which is the smallest administrative district level in Ethiopia, in four regions (Amhara, Southern Nations, Nationalities, and Peoples′ Region (SNNPR), Oromiya, and Tigray) and to incorporate defined methods, indicators, and research techniques to include both WASH and nutrition data. Variations between sub-regions are noted in overall rates of stunting; however, the further analysis of variations and differences at a sub-regional level was identified as beyond the scope for this specific study. As this investigation was part of a wider intervention-based study, the selection of the sub-regions was conducted for the purpose of delivering the intervention across the intervention and control groups. See Table 1 for an illustration of the number of kebeles per sub-region that were selected for the intervention and control group (see Table 1). 

The results of this paper draw upon the baseline data collected between June and August 2013. As this study does not examine the impact of the interventions, the end-line data was not included in this paper. The sample focuses on individual-level data for children under five years of age, and household-level data in order to capture factors related to water, sanitation, weight, age, and the height of children.

### 2.1. Data Set and Sampling

The subset of data used for this study is drawn from a baseline survey from 3200 households from 80 different kebeles, the smallest administrative unit in Ethiopia, using a cross-sectional methodology [1]. The baseline dataset was used to provide a snapshot of the current state of factors and their association with stunting. The study sample was drawn from each of the regions: Amhara, Oromiya, SNNPR, and Tigray. The target population was children under five residing within the selected households to be assessed on nutritional status. Mothers/caretakers of children under five were also interviewed for an assessment of knowledge and practice of good nutrition and health behaviours. As this study includes a representative sampling strategy to reflect the characteristics and differences across administrative units and the aims of the wider study, the distribution of the sample from the different regions is shown below with type of intervention received in each kebele indicated in Table 1. While this study does not include the intervention or end-line data, it is important to take this into account, as it was a factor influencing the sample design. This sample was drawn from the wider program of work overseen by UNICEF. Four category groups were assigned, which include a control group (1600) and three groups receiving different community-based interventions in hygiene promotion, WASH, and MUS (1600 in total).

The 80 kebeles (40 each from the intervention and control sites) were sampled using the proportion of the intervention groups for each of the regions (Amhara, Oromiya, SNNPR, and Tigray). Each kebele consists of at least 500 families. The cluster groupings of kebeles (woredas) include 30 intervention and 92 control groups in the four regions, which were purposefully selected by UNICEF based on their funding mechanism by either the Government of Netherlands or the Canadian International Development Agency. For the 30 intervention woredas, UNICEF provided a list of 576 kebeles that formed the basis for sampling. The sample was divided into 40 clusters and a weighted percentage was attributed to each of these three different interventions. Based on the proportion of kebeles in each intervention group (40% with CWS, 55% with CH&S, and 5% with MUS) and the proportion of kebeles in each region (34% in Amhara, 14% in Oromiya, 34% in SNNPR, and 18% in Tigray), the number of kebeles required for the sampling was determined. For the 92 control woredas, there is a list of 2158 kebeles that formed the basis for sampling. The number of kebeles to be sampled per region was determined using the same proportional distributions as the intervention groups (34% in Amhara, 14% in Oromiya, 34% in SNNPR, and 18% in Tigray). 

Sampling was conducted in consultation with the village leader to determine households with children. The sampling interval was determined by dividing the total number of households with a child by the number of households to be interviewed. Then, the team selected a random number between one and the sampling interval. This random number indicated the first household to be surveyed. The team interviewed the household administering the WASH household questionnaire, including any children under 5 and their mothers/caregivers. Then, the team moved to the next random household (by adding the random number to the sampling interval). Data collection continued in this way until the village boundary was reached (Subsequent houses were selected by going back to the center of the village and continuing the selection of every random household along the same line in the opposite direction until the kebele boundary was reached). As necessary, the team would return to the center, take the 90-degree line from the first line, and continue to sample the random households, repeating again as necessary. If a member of the household was not present, the team would go back later and continue to visit the household until the team left the area. If the household could not be interviewed, the household would be replaced by another by continuing the sampling method.). The team counted the households in the selected direction starting with one and moving until the house with the selected random number was reached.

### 2.2. Validity and Reliability Considerations and Mitigation

UNICEF was concerned about the enumerator effect, which can possibly result in biased data collection. Hence, for each questionnaire administered, the study included a double data collection approach. For each interview conducted, two enumerators separately recorded the data obtained in two separate questionnaires, such that each interview would have two separate completed questionnaires. After every third interview conducted by a pair, enumerators were rotated so that different pairings were formed. This allowed the data obtained by each enumerator to be individually analyzed, compared for percent agreement, and provided opportunities to identify if there was one or more poorly performing enumerators. 

To address potential recall and response bias, responses were triangulated to identify inconsistent patterns. Age of children was recorded in months, while for women it was recorded in years. For many rural households, it is often difficult to accurately assess the age of children. Where a birth certificate or vaccination card was not available for the child and/or the mother is unsure of the child’s age, the field team members discussed with the mother events that happened around the birth of their children. With this information, the enumerators linked the responses to a pre-prepared calendar of local events to work out the age of the child. 

Age heaping refers to the generalization of specific ages into preferable ages by respondents. Age heaping is often caused by the respondent being unsure of their exact age, reluctant to provide it, and/or where there is a cultural preference for certain numbers—for example, those ending in ‘0′ or ‘5′. Age heaping is often higher where there are high levels of illiteracy and where the recording of births is not common practice. In Ethiopia, age heaping is most likely caused by a respondent being unaware of their child’s exact age, and subsequently generalizing to a number that feels plausible. This has significant implications for determining the correct nutritional status of children in this study. However, there are ways of minimizing the problem, most of which involve increasing the specificity when requesting the age or working through proxies, including data on age being derived from answers to a two-part question (i.e., age and date of birth) to minimize the effect of age heaping. 

As the most reliable evidence, research teams requested to see the birth certificate of the child, or the child’s baptized date for the households of Orthodox followers; where these were not available, the research teams requested the child’s vaccination cards, which should indicate the date of birth, or at least the date of first injections. From these documents, the field team tried to work out and record the age. Where neither of these official documents were available, proxies such as determining time-related data from a local calendar of events was used. Overall, local language speakers formed part of the field teams to help estimate age accurately. The field staff were trained to elaborate the respondent’s answer to as specific a date as possible. If the mother replied that they gave birth around a festival, for example, the interviewer will ask them to remember if it was before, during, or after the festival, and its relative closeness to the proceeding or succeeding events.

While the study does not present a baseline evaluation or monitor the performance of the program itself, the data collection is embedded in the context of an integrated approach to tackling stunting.

### 2.3. Survey and Anthropometric Instruments

The survey instrument included a WASH and stunting questionnaire administered in every one of the 40 intervention and the 40 control kebeles. Measurements used to assess stunting included anthropomorphic outcome variables such as weight, height, and age. To assess associations between stunting and WASH factors, z-scores are used to generate a linear regression model that represents these associations using an F-test for goodness of fit. The authors acknowledge that depending on which reference is used, in this case the WHO reference, the stunting rate varies. While this variation is not ideal, the benefit is that it does allows for comparison with a wider range of studies, as the WHO reference is commonly utilized. The authors also acknowledge that there are limitations to applying the WHO reference in developing countries. This is taken into consideration; however, for the purpose of standardization and potential comparison with other countries, the WHO reference is used. 

For anthropometric measurements, the team used the accepted standard-length boards, SECA digital scales, and iodine testing kits supplied by UNICEF. To ensure validity and reliability, the field teams verified the proper functioning and calibration of the measurement instruments before the commencement of fieldwork and on a daily basis before starting their activities. The necessary skills and procedures for the calibration of these instruments was part of specific modules of the field supervisors’ and data collectors’ training. Instruments that were found to be malfunctioning or unreliable were fixed, if possible, or replaced.

Accompanying the household questionnaire, the anthropometric survey was conducted among children under five and women of childbearing age to determine the levels of underweight, wasting, and stunting. The following information was collected through anthropometric measurements:

1. Height: Length of infants (0–23 months) was measured in a recumbent position to the nearest 0.1 cm using a locally-made board with an upright wooden base and a movable headpiece. The height of children in the 24–35 months age range was measured in a standing, upright position to the nearest 0.1 cm using a locally-made vertical board with a detachable sliding headpiece.
2. Weight: Children were weighed with light clothing and without shoes. In order to weigh children under the age of 3 years, the UNISCALE method was employed using the UNICEF mother/child electronic scale, which requires the mother and child to be weighed simultaneously. (This process entails the following activities: 1. Asking the mother to stand on the scale 2. Recording the weight and including the reading with one decimal point (e.g., 65.5 kg). 3. Passing the child to a person nearby. 4. Recording the second reading with just the mother (e.g., 58.3 kg). 5. Noting the difference (e.g., 7.2 kg) is the weight of the child.).

To ensure the validity and reliability of the anthropometric measurements, checks were incorporated into the process (see Appendix A). Key checks included minimizing clothing on the child, ensuring the scale is not over-heated in the sun and is placed on an even surface, and calibration checks conducted for the scale after each household. 

Stunting estimations were derived using IBM SPSS Statistics software calculated using a WHO anthropometric macro [40] that compares children’s height to the reference population, which allows z-scores to be calculated directly into the dataset. The z-score system defines an anthropometric value as a number of standard deviations above or below the reference mean or median value. The equation used for calculating the z-score is [2]:

Stunting Z-score (or SD-score) = (Observed value − Median value of the reference population)/(Standard deviation value of the reference population)

Children are considered stunted when below −2 standard deviation (SD) and heavily stunted when below −3 from median height for age (HAZ) of a reference population. 

### 2.4. Regression Models

A combination of linear and hierarchical regression models was used to explore the determinants of stunting and the impact of WASH on stunting. Linear models helped to establish associations in an area where there was currently, at the time, limited study in the region. Hierarchical regression analyses were subsequently used to examine changes in association as more potential confounders were taken into account. There are other modeling approaches that were considered to detect more complex relationships such as logistic regression; however, in a relatively understudied area, early cross-tabulations between stunting and predictors provided a starting point for detecting linear relationships and for further exploration. Linear regression models first assess the strength of the association between stunting and individual factors such as age of the child, age category of the child, gender of the child, and gender of the caregiver. 

The models presented in the results include single regression analyses and the hierarchical regression analyses. The linear models were used to inform understanding of the variance of each predictor before including in a combined regression model. See Appendix B for a description of how multicollinearity and heteroscedasticity were addressed. A hierarchical regression model was built for the combined variables, bearing in mind the associations found in the single regressions and adding them in sequence. Each step of the analysis adds one or various predictors to understand their variance individually. This is done to enable the comparability of models as additional factors of interest are added. For example, as each variable is added, the authors noted any significant changes by adding: Caregiver Gender, then Caregiver Gender and Age Category, Caregiver Gender, Age Category and WASH Factors, and so on and so forth. 

## 3. Results

The total number of children under five years of age with available data was 2400, with 1200 children (the sample size was determined to allow a 10% change in stunting prevalence in children 0–59.9 months over the period of the intervention using the following formula n = D [(Zα + Zβ)2 * (P1 (1 − P1) + P2 (1 − P2))/(P2 − P1)2], where D, the design effect = 2, P1, the current level of stunting = 0.44, and P2, the expected level of stunting = 0.34, Zα, the confidence level = 1.645, and Zβ, the confidence level for power = 1.645.) within each arm of the study to permit the detection of stunting and diarrhea across the four regions of Amhara, Oromiya, SNNPR, and Tigray. Table 2 shows the observed stunting rates (percentages) across the dataset. The observations are disaggregated in terms of gender and age groups. The total rate of stunting is 45.7% (SD 1.53, mean −1.82).

**Figure 1:**
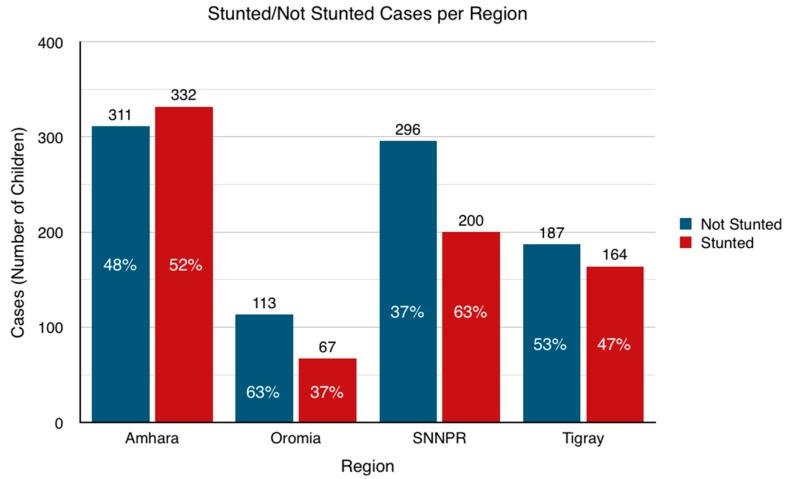
Stunting Rates for Children <5 Years Old in Different Regions.

The total stunting rates for boys and girls vary by 3.3%, with a total rate of 45.7% in the sample population (SD for gender 0.50, mean 1.49). Boys younger than 6 months old generally have lower stunting rates in comparison to girls. However, the trend changes after 6 months old, with 6% and 3.1% more stunting rates in age category 2 and 3, respectively. It is important to note that the total stunting rates increase with age, starting at an average of 27.5% in the first age category, then increasing up to 51.5% between 24–59 months old. Therefore, it is important to underline that the overall stunting rate in the country is most probably higher than the average total of 45.7% detailed, which represents the average for children younger than five years old. There is some variation across the regions, as shown in Figure 1. A full list of all predictors considered is available in Table 3.

There is evidence to suggest that the variance in stunting prevalence is significant across regions (Pearson chi-square *p* < 0.0001). This suggests that socioeconomic, environmental, and nutritional factors vary from one region to another, and that there is a correlation between region and stunting rate in this context. Oromiya shows evidence of lower proportions of children under five who are stunted, as compared with the other regions. SNNPR contains higher proportions of children who are stunted.

**Figure 2:**
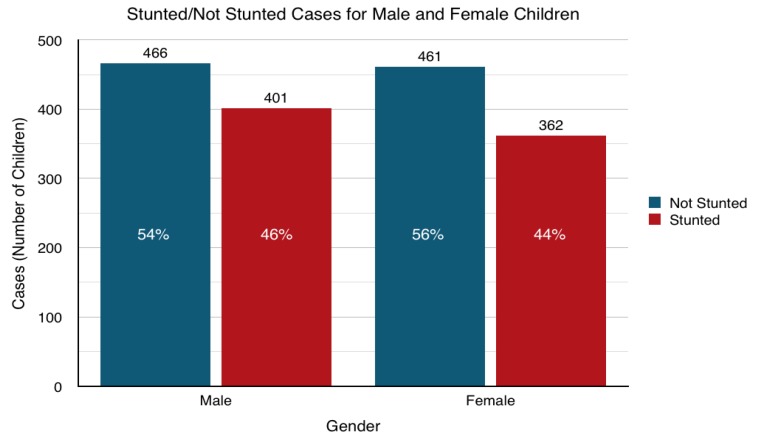
Stunting Rates for Children <5 Years Old across Gender.

There was strong evidence to suggest the variance in stunting prevalence is not significant across gender (Pearson chi-square *p* = 0.168), as illustrated in Figure 2. This is an important result to take into consideration as a descriptor, but will be further tested in linear regression for confirmation.

**Figure 3:**
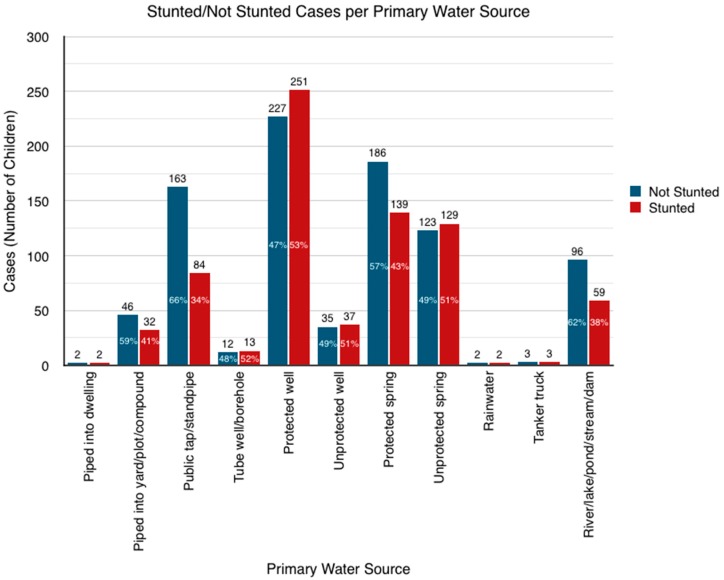
Stunting Rates for Drinking Water Source.

There is evidence to suggest that the variance in stunting prevalence is significant across primary water source (Pearson chi-square *p* < 0.0001), as shown in Figure 3.

### 3.1. Associations with Stunting

To determine whether there is an association between WASH and stunting, single regression analyses is utilized to identify strong associations. These findings provide the basis for deriving a combined model that also accounts for collinearity in its derivation. Table 4 shows the effect of each predictor on stunting. 

Caregiver Gender and Age Category are also found to have strong associations with stunting. The following WASH factors demonstrate strong associations with stunting prevalence: drinking water sources, sanitation facilities (children), handwashing before eating (mothers and children), handwashing after defecation (mothers), handwashing with water (mothers and children), and handwashing with water, soap, or ash (mother). Water sources for other household purposes are omitted from the subsequent hierarchical regression model because of high collinearity with drinking water source. The variable representing sanitation facilities for children below five has been included in the hierarchical regression model, as it is the factor with high influence. 

After examining associations using linear regression and identifying evidence for collinearity, washing practices for men, women, children below five, and children above five, three variables were noted to not be statistically significant, which include latrine location, child feces disposal, and handwashing after defecation (children). Mothers handwashing before eating was removed from the overall model, as it was highly variable with children below five handwashing before eating (and had a lower R^2^ value). 

### 3.2. Combined Model

After removing statistically non-significant predictors and checking for collinearity, Caregiver Gender, Age Category, and the following WASH variables are used for the hierarchical regression analysis: drinking water source, handwashing after defecation (mothers), and handwashing before eating (children <5). Table 5 shows the model with the demographic predictors (Model 1) and the combined model (Model 2), and includes the strength of the association (*P*) for the combination of predictors.

## 4. Discussion

This paper analyses significant determinants of stunting for children under 5 years of age in the context that emerges from findings from this study in rural Ethiopia and the existing body of literature both in and outside of Ethiopia. WASH factors that feature in this study include handwashing, drinking water facilities, and wider determinants, including children’s age, and the gender of children and caregivers. 

Age was found to have the strongest correlation to stunting in this study group. This is to be expected, as the symptoms of stunting appear after prolonged inadequate environment and nutrition. These results are consistent with the literature, and strengthen the consensus that earlier interventions in a child’s life are important. While many factors may influence this correlation, the literature suggests improved behavioural practices such as handwashing before eating early on may be important to consider along with other factors closely associated with the age of the child. At the sub-regional level within the region studied, Oromiya had lower levels of stunting proportionally compared with the other sub-regions (Amhara, SNNPR, and Tigray). Although this analysis goes beyond the scope of the paper, there is a recognition that this is a limitation, and would require further analysis as to the factors that would render this difference. 

In this study, the gender of the primary caregiver is noted to be a statistically significant predictor of stunting prevalence with the presence of a male caregiver implying a lower prevalence of stunting. This may be explained by a trend that the presence of a male caregiver, in addition to a female caregiver, would suggest overall more resources. However, this social factor only explains 0.6% of the stunting prevalence in this dataset. This result is consistent with another study in Ethiopia where female-headed households had a higher prevalence of stunting in children, which was attributed to a lack of access to livelihood opportunities [30]. With acknowledgement of these livelihood opportunities, an explanation not explored in this study is that the gender of the primary caregiver may have collinearity with other factors such as socioeconomic status (as a higher prevalence of stunting in children to female-headed households was attributed to a lack of access to livelihood opportunities). Further investigation should also explicitly explore differences between female-headed households and other household compositions. 

Overall, the WASH determinants in this sample account for approximately 7% of the variation in stunting. In isolation, this is not the large share of stunting; however, if taken into consideration alongside other nutrition factors such as the quality and quantity of food, addressing structural determinants such as water source and behavioural practices may provide opportunities to have a stronger, more targeted integrated approach to stunting prevention. 

With these factors in mind, the WASH determinants of interest for stunting in this study are drinking water source, handwashing after defecation (mothers), and handwashing before eating (children). The association with handwashing after defecation can be explained by the fact that women would be the primary handler of the child, and any contamination would likely be passed to the child. For children, handwashing before eating is associated with stunting namely as children would be ingesting unclean water and/or other pathogens. There were several factors such as other household water uses and different uses of soap and water that are significant in the single linear regression; however, these had strong collinearity with factors such as drinking water source (other household water uses) and handwashing with soap (handwashing after defecation). 

Sanitation may have secondary importance as sanitation facility i.e., faeces disposal, was found to be non-significant. There is an argument that handwashing overall may nullify the effect of sanitation facility, yet likely with some limitations. This suggests that there may be a greater need to prioritize support for good handwashing and hygiene practices in locations with poor-quality sanitation facilities. This finding clashes with the finding of the Ahmadi 2018 study, which identified open defecation, along with child’s sex, age, and region as strongly correlated with stunting [36]. A reason for this difference is that waste disposal and open defecation and its consequences are quite context-specific, and may vary considerably. What this shows is that there is a need for a harmonization of factors, and greater diversity in understanding how these factors are measured and accounted for. 

Handwashing for both children under five and their mothers initially seems to indicate higher rates of stunting with the inclusion of soap not being a stronger influence. A possible explanation for this is that soap is most effective with clean drinking water. Where access to a clean drinking water source is unavailable, the benefits of soap may not be realized. This may indicate the value added by access to clean water and hygiene and handwashing practices in communities with unsafe sanitation facilities. However, it is remiss to not acknowledge that handwashing tends to be over-reported by survey participants (desirability and recall bias). Taking this finding into account, the overestimation of the results of handwashing and its linkage to stunting also needed to be taken into account. Efforts by UNICEF as described in Appendix A did introduce rigor to the study design in order to reduce bias; however, overall over-reporting of handwashing may not have been eliminated entirely. 

These findings are consistent with literature on the integration of WASH in the prevention of stunting, particularly in reframing undernutrition to include more than food access, quantity, and quality/diversity and to consider broader environmental factors [11,17]. The multi-village study from Ethiopia discussed earlier in our paper noted that WASH interventions that included improved access to WASH services and improved maternal knowledge on hygiene practices in 11 villages in Ethiopia were linked to a significant reduction in stunting [34]. The findings of this study contribute further detail to the specific WASH activities that help to better contextualize the timing of handwashing by children and caregivers and source of water.

Of the demographic and descriptive determinants, child gender is found to be a non-significant predictor of stunting for this study group. The literature on this is mixed, with some studies indicating that child gender has a role to play [21,41], although these studies are not specific to Ethiopia or Africa more broadly, as both studies emerge from the South Asian context. For Ethiopia, there are studies indicating the significance of gender of child for stunting [25,35] versus studies placing more prominence on livelihood and access to farming opportunities in rural communities [42]. This implies that the strength of the association between stunting and gender may be highly dependent on other contextual and behavioural factors. 

These findings contribute an evidence base to inform further exploration of linkages between WASH and stunting in the Ethiopian context. Recommendations that emerge from these findings are to reduce the effect of stunting through the promotion of safe drinking water sources and handwashing practices with greater attention to clean water sources, and promoting the use of handwashing after defecation for mothers and before eating, especially for children where there is access to clean drinking water. 

The strength of this study is the provision of context-specific evidence for associations with stunting that explore specific WASH factors in a context where this has not been explored at this scale. These findings are integrated with demographic and social factors that affect health, which together provide evidence for an integrated approach to tackling the challenge of stunting. This study also draws upon a relatively large sample size for the scale of the country, which suggests that future studies will also need to ensure large enough population samples to have the numbers for establishing claims on whether factors such as livelihood opportunities, the education levels of parents/caregivers, and gender can influence stunting.

This study provides an overview of social and WASH determinants for stunting in Ethiopia. With these contributions, there are limitations of this study that should be taken into account for future work. A limitation of this study is the exclusion of end-line data from the analysis. The baseline data in this paper does not provide evidence on stunting and determinants over a period of time, and does not test the value added by UNICEF’s WASH intervention, which was conducted after the collection of the baseline data. In terms of variation by region, the authors acknowledge in the limitations that the differences in regions, how they contrast, and what can be learnt should be explored in further research, although this went beyond the scope of this paper. The sub-regional differences acknowledged between Oromiya for example, which had proportionally lower numbers of children who were stunted, could be explored in an analysis that potentially could isolate risk factors that may be related to demographics, behaviour, and/or a combination of the variables explored here. This is an area of extreme importance for delivering locally tailored interventions; however, for the purpose of this study, this remained at a regional level. Contextual factors such as differences in toilet coverage and quality, population density, humidity, and/or dryness need to be explored in tandem with demographic factors such as ethnicity and religion, as they could also be highly correlated with socioeconomic status. 

With respect to the over-reporting of handwashing by survey participants, greater efforts should be taken to ensure that measuring behaviour such as handwashing is confirmed through a triangulation of different methods. Future work should consider to what extent wealth and an asset base have an impact on households, especially within vulnerable groups and communities. As noted in the methods, biases such as recall bias, particularly in providing health data related to age, requires planning at the fieldwork stage and robust methodologies such as the one used by UNICEF (Appendix A). There are also limitations in using the WHO reference for assessing the growth of Ethiopia’s children as well as children in developing countries more broadly. This is acknowledged in the methods and is reiterated here with a view that further research might consider other alternatives. 

Areas for further research include exploring the contextual factors, environmental factors, and socioeconomic factors described in the limitations along with sub-regional differences. Exploring fully the collinearity between factors such as the gender of the caregiver and socioeconomic status, for example, may also provide additional insight. Future studies could include analysis of other factors such as livelihood opportunities, the education levels of parents/caregivers, and gender. Examining this in the context of the regional, local differences, cultural and ethnic variations would also lend greater granularity in order to adapt interventions to localized contexts. More broadly, these findings from a research perspective also suggest the need for interdisciplinary research for public health and environmental research agendas, which can improve the overall sustainable development for households through integrated perspectives. 

## 5. Conclusions

Stunting is complex and influenced by multiple factors and structural determinants such as caregiver gender, age, water source, and behavioural practices around handwashing. Demographic and social factors such as the age of the child and gender of the caregiver also contribute to the reality that stunting is complex. While eliminating stunting will not be addressed through tackling WASH factors alone, what this study provides is a starting point for exploring WASH factors in the Ethiopian context and evidence for including WASH factors within an integrated approach to tackling stunting. The study provides programmatic recommendations that include: (1) strengthening efforts to improve handwashing behaviour for mothers and children; (2) prioritizing access to clean water sources; and (3) supporting specific WASH activities that help to better contextualize the timing of handwashing by children and caregivers, and source of water. More broadly, the study is consistent with evidence in the literature to reframe undernutrition to include more than food access, quantity, and quality/diversity, and consider broader environmental factors.

This study indicates consistency with recommendations for integrated interventions that improve feeding practices, hygiene behaviours, and the enabling conditions and to embed WASH within a holistic, integrated approach to tackling stunting. This study demonstrates the need for interdisciplinary research at scale to develop joint health, education, and behavioural change interventions that improve feeding practices and offer a holistic, integrated approach to tackling stunting by addressing the environmental factors and designing improvements to address them.

## Figures and Tables

**Table 1:** Distribution of sample by region and type of intervention.

Region	Type of Intervention				Control
	CBN &CWS	CBN & CH &S	CBN & MUS	Total	CBN
Amhara	8	4	1	13	13
Oromiya	3	3	0	6	6
SNNPR	4	9	1	13	13
Tigray	1	6	7	7	7
Total	16	22	2	40	40

The interventions referred to are as follows: CBN = Community-Based Nutrition, CWS = Community Water Supply, CH & S = Community Hygiene & Sanitation, and MUS = Multiple Use Services.

**Table 2:** Stunting rates against age groups.

Stunting Rates			
Age Group	Boys	Girls	Total
0–5 months	27.63%	30.99%	29.30%
6–23 months	41.83%	36.55%	39.20%
24–59 months	52.88%	49.50%	51.20%
Total	47.30%	44.00%	45.70%

**Table 3:** Predictors of stunting (standard deviation SD and mean).

Predictors	SD	Percentages
Gender of caregiver	0.278	92% Female 8% Male
Gender of child	0.50	49% Female 51% Male
Age category	0.67	10% 0–5 months 31% 6–23 months 59% 24–59 months
Drinking water source	2.61	
		0.2% Piped into dwelling 5% Piped into compound 15% Public tap/standpipe 2% Tube well/borehole 29% Protected well 4% Unprotected well 20% Protected spring 15% Unprotected spring 0.2% Rainwater 0.4% Tanker truck 9.4 River/lake 1.4% Other
Water source for other household purposes	3.09	
		0.1% Piped into dwelling 5% Piped into compound 13% Public tap/pipe 2% Tube well/borehole 23% Protected well 8% Unprotected well 15% Protected spring 14% Unprotected spring 0.3% Rainwater 0.2% Tanker truck 20% Cart with small tank 0.1% River/lake 0.6% Other
Sanitation facility (Mothers)	0.134	
		0.1% Flush to septic tank 0.1% Flush to pit latrine 0.3% Flush to somewhere else 33% Don’t know 28% Ventilated improved pit latrine 10% Pit latrine with slab 5% Pit latrine without slab 6% Pit latrine with conventional material 17% Composting toilet 0.1% Ecological toilet 0.1% Bucket toilet 0.1% Hanging latrine 2% No facility, bush/field farm
Sanitation facility (Children <5)	11.108	
		0.1% Flush to septic tank 0.2% Flush to pit latrine 0.1% Flush to somewhere else 15% Don’t know 8% Ventilated improved pit latrine 5% Pit latrine with slab 1% Pit latrine without slab 19% Pit latrine with conventional material 12% Composting toilet 36% Ecological toilet 0.9% Bucket toilet 0.1% Hanging latrine 2% No facility, bush/field farm
Latrine location	0.385	
		1.5% At the house 93% At the compound 4% At the neighbor’s compound 1.5% Within the area
Child feces disposal	1.76	
		2% Children use the latrine 59% Disposed at latrine 1% Disposed in a ditch 11% Disposed with the garbage 1% Buried 26% Left at the open air
Handwashing before eating (Mothers)	0.117	1% No 99% Yes
Handwashing after defecation (Mothers)	0.343	14% No 86% Yes
Handwashing before eating (Children)	0.491	41% No 59% Yes
Handwashing after defecation (Children)	0.479	35% No 65% Yes
Use of soap after defecation	0.423	23% No 77% Yes
Use of soap before eating	0.296	10% No 90% Yes
Handwashing with soap or ash and water (children)	0.065	99% No
		1% Yes
Handwashing with soap or ash and water (mother)	0.139	98% No 2% Yes
Handwashing water only (children)	0.50	51% No
		49% Yes
Handwashing water only (mother)	0.494	79% No 21% Yes

**Table 4:** WASH Determinants: Single Regression Analyses.

Outcome	Predictors	*p*	*r* ^2^
Z-Score	Drinking Water Source	<0.001	0.020
Z-Score	Water Source for Other Household Purposes	0.01	0.021
Z-Score	Sanitation Facility (Mothers)	0.134	0.01
Z-Score	Sanitation Facility (Children <5)	0.048	0.016
Z-Score	Latrine Location	0.567	0.006
Z-Score	Child Feces Disposal	0.659	0.392
Z-Score	Handwashing Before Eating (Mothers)	0.024	0.003
Z-Score	Handwashing After Defecation (Mothers)	0.010	0.004
Z-Score	Handwashing Before Eating (Children)	0.0001	0.01
Z-Score	Handwashing After Defecation (Children)	0.202	0.001
Z-Score	Use of Soap After Defecation	0.116	0.001
Z-Score	Use of Soap Before Eating	0.08	0.002
Z-Score	Handwashing with Soap or Ash and Water (children)	0.285	0.001
Z-Score	Handwashing with Soap or Ash and Water (mother)	0.006	0.005
Z-Score	Handwashing Water Only (children)	0.0001	0.014
Z-Score	Handwashing Water Only (mother)	0.0001	0.014

**Table 5:** Combined Models: Hierarchical Regression Analysis for Social and WASH Determinants.

Outcome	Predictors	*p*	*r* ^2^
Z-Score	**Model 1** Caregiver Gender (SD 0.278) Age Category (SD 0.671)	<0.001	0.059
	**Model 2** Caregiver Gender Age Category Drinking Water Source Hand Washing After Defecation (Mothers) Hand Washing Before Eating (Children < 5)	<0.001	0.072

## Appendix A

### Appendix A.1. Data Quality Assurance Procedures

**Table A1:** Data Quality Assurance Procedures.

Data Aspects	Data Quality Assurance Procedures
Data Instruments	The data collection instrument should be designed carefully to capture the intended information. The tool should be well understood by the enumerators. The appropriateness of the tools needs to be pretested.
Quality Control during Data Collection	Completeness of all study forms will be checked by the supervisors at all survey sites. All questionnaires will be reviewed for completeness and/or errors on a daily basis by the supervisors.
Data Entry and Back-Up	Questionnaires will be transferred from the field to the headquarters for data entry, back-up, and storage. Each questionnaire will be entered separately and independently such that comparison between each of the questionnaires obtained from the same respondent will be possible. A computerized database storage has been developed using appropriate software. The database and the computer holding the database will be password-protected; the data entry clerks will only have data-cleaning privileges, whereas the data manager will have full access to the server for data correction and cleaning. Electronic data checked for errors, and extreme values will be corrected by data clerks through the editing guidelines provided by the data manager. Data will be backed up by the data manager on the server and by external hard disk drive with full application programs. The back-up external hard disk drive data will be stored in a fire-proof safety cabinet in the IT department, and an external copy will be kept in a separate office in CD-R format.
Data Validation	The data manager will check for consistency between each enumerator for the same respondent, providing a percent agreement for every single pair of enumerators The data manager will use frequency checks of indicators to examine the database entries for clear documentation and identifying data outliers on a daily basis.
	The data manager will double-check 10% of the entered data.
	The data manager will send query reports about the exemptions and errors found in database entries to the survey team for decisions.
	Database entry will consist entirely of numeric data that don’t contain personal identifiers.
	Analyses and reports will not contain personal identifiers, and results will be reported in aggregates.

### Appendix A.2. Anthropometric Measurements

The length of infants (0–23 months) was measured in a recumbent position to the nearest 0.1 cm using a locally made board with an upright wooden base and a movable headpiece. The height of children in the 24–59-month age range was measured in a standing, upright position to the nearest 0.1 cm using a locally made vertical board with a detachable sliding headpiece.

Anthropometric indices were calculated, as per WHO child growth standards, using the age, height, and weight data collected on all children under the age of five. Baseline estimates were calculated for height-for-age (HAZ), weight-for-age (WAZ), and weight-for-height (WHZ) standard deviations. Prevalence of stunting (defined as HAZ < −2 z-scores), underweight (defined as WAZ < −2 z-scores), wasting (defined as WHZ < −2 z-scores), and overweight (defined as WHZ > 2 z-scores) were also assessed.

The F test of goodness of fit’s outcome values of *p* < 0.05 were considered statistically significant at the 5% significance level [32]. In this study, the null hypothesis was therefore systematically rejected at the 5% significance. This F test was used to understand if the different categorical predictors analyzed showed significant variance in stunting rates. Contingency tables and graphs were developed to visualize the distribution of the data according to various variables, and the test was performed in order to see if the variation in the data was statistically significant or purely up to chance. These observations were useful in obtaining an understanding of the significant social and WASH variables before undertaking regression analyses.

For population-based assessment, the z-score is widely recognized as the best system for analysis of length/height data.

## Appendix B

Multicollinearity was double-checked by variance inflation factors (VIF) and collinearity values for each categorical variable in a combined regression model. VIF <3 is generally considered safe in terms of collinearity [33], and was therefore used in this analysis as the threshold for including variables in the combined models. Collinearity checks were positive as all VIF values were largely below 3, indicating that there was no multicollinearity in the models. Histogram and P-P plots were used to confirm the validity of the analysis.

Heteroskedasticity was controlled for by visual inspection of scatter plots, as well as running the Breusch–Pagan and Koenker tests for heteroskedasticity with an SPSS macro code [43]. Then, their statistical significance allowed to decide whether to re-estimate the regression models with heteroskedastic consistent standard errors and significance *p*-values or not.

## References

[1] Shields K. (2015). Wet Nutrition 2013 Baseline Survey Report.

[2] World Health Organisation (WHO) (2016). WHO Global Database on Child Growth and Malnutrition. http://www.who.int/nutgrowthdb/about/introduction/en/index2.html.

[3] European Parliament (2015). The Social and Economic Consequences of Malnutrition in ACP Countries (Background Document). http://www.europarl.europa.eu/meetdocs/2009_2014/documents/acp/dv/background_/background_en.pdf.

[4] Smith L.C., Haddad L. (2015). Reducing child undernutrition: Past drivers and priorities for the post-MDG era. World Dev..

[5] Black R.E., Victora C.G., Walker S.P., Bhutta Z.A., Christian P., de Onis M., Ezzati M., Grantham-McGregor S., Katz J., Martorell R. (2013). Maternal and child undernutrition and overweight in low-income and middle-income countries. Lancet.

[6] UNICEF, World Health Organisation (WHO) and World Bank (2017). Child Malnutrition Estimates. http://www.who.int/nutgrowthdb/estimates2016/en/.

[7] International Food Policy Research Institute (2015). Global Nutrition Report 2015—Africa Brief, Actions and Accountability to Advance Nutrition and Sustainable Development. https://www.globalnutritionreport.org/files/2015/11/GNR2015-Africa-Brief1.pdf.

[8] African Union Commission, NEPAD Planning and Coordinating Agency, UN Economic Commission for Africa, UN World Food Programme (2014). The Cost of Hunger in Africa: Social and Economic Impact of Child Undernutrition in Egypt, Ethiopia, Swaziland and Uganda.

[9] UNICEF (2013). Improving Child Nutrition. The Achievable Imperative for Global Progress. United Nations Children’s Fund (UNICEF). https://www.unicef.org/gambia/Improving_Child_Nutrition_-_the_achievable_imperative_for_global_progress.pdf.

[10] WHO (2019). WHO Conceptual Framework on the Context, Causes and Consequences of Childhood Stunting. https://www.who.int/nutrition/healthygrowthproj/en/index1.html.

[11] Chambers R., von Medeazza G. (2014). Reframing Undernutrition: Faecally-Transmitted Infections and the 5 As.

[12] Huicho L., Huayanay-Espinoza C.A., Herrera-Perez E., Segura E.R., de Guzman J.N., Rivera-Ch M., Barros A.J.D. (2017). Factors behind the success story of under-five stunting in Peru: A district ecological multilevel analysis. BMC Pediatr..

[13] Wolf J., Prüss-Ustün A., Cumming O., Bartram J., Bonjour S., Cairncross S., Clasen T., Colford J.M., Curtis V., De France J. (2014). Systematic review: Assessing the impact of drinking water and sanitation on diarrhoeal disease in low-and middle-income settings: Systematic review and meta-regression. Trop. Med. Int. Health.

[14] Humphrey J.H. (2009). Child undernutrition, tropical enteropathy, toilets, and handwashing. Lancet.

[15] Cumming O., Cairncross S. (2016). Can water, sanitation and hygiene help eliminate stunting?. Curr. Evid. Policy Implic. Matern Child Nutr..

[16] Val C., Cairncross S. (2003). Effect of washing hands with soap on diarrhoea risk in the community: A systematic review. Lancet Infect. Dis..

[17] Budge S., Parker A.H., Hutchings P.T., Garbutt C. (2019). Environmental enteric dysfunction and child stunting. Nutr. Rev..

[18] Lee C., Lakhanpaul M., Stern B.M., Parikh P. (2019). Associations between the household environment and stunted child growth in rural India: A cross-sectional analysis. UCL Open.

[19] Spears D., Ghosh A., Cumming O. (2013). Correction: Open Defecation and Childhood Stunting in India: An Ecological Analysis of New Data from 112 Districts. PLoS ONE.

[20] Seedhom A.E., Mohamed E.S., Mahfouz E.M. (2014). Determinants of stunting among preschool children, Minia, Egypt. Int. Public Health Forum.

[21] Mahmood M.A., Nasir Z.M. (2001). Determinants of Growth Retardation in Pakistani Children under Five Years of Age [with Comments]. Pak. Dev. Rev..

[22] Aklima J., Yamamoto S.S., Malik A.A., Haque M.A. (2011). Prevalence and determinants of chronic malnutrition among preschool children: A cross-sectional study in Dhaka city, Bangladesh. J. Health Popul. Nutr..

[23] Smith L.C., Kahn F., Frankenberger T.R., Wadud A. (2013). Admissible evidence in the court of development evaluation? The impact of CARE’s SHOUHARDO Project on child stunting in Bangladesh. World Dev..

[24] Vollmer S., Bommer C., Krishna A., Harttgen K., Subramanian S.V. (2016). The association of parental education with childhood undernutrition in low-and middle-income countries: Comparing the role of paternal and maternal education. Int. J. Epidemiol..

[25] Teshome B., Kogi-Makau W., Getahun Z., Taye G. (2009). Magnitude and determinants of stunting in children underfive years of age in food surplus region of Ethiopia: The case of west gojam zone. Ethiop. J. Health Dev..

[26] United Nations Standing Committee on Nutrition (2010). Progress in Nutrition: 6th Report on the World Nutrition Situation. https://www.unscn.org/files/Publications/RWNS6/report/SCN_report.pdf.

[27] World Health Organisation (WHO) (2014). WHO Global Nutrition Targets 2025: Stunting Policy Brief. http://apps.who.int/iris/bitstream/10665/149019/1/WHO_NMH_NHD_14.3_eng.pdf?ua=1.

[28] Megabiaw B., Rahman A. (2013). Prevalence and determinants of chronic malnutrition among under-5 children in Ethiopia. Int. J. Child Health Nutr..

[29] Geberselassie S.B., Abebe S.M., Melsew Y.A., Mutuku S.M., Wassie M.M. (2018). Prevalence of stunting and its associated factors among children 6-59 months of age in Libo-Kemekem district, Northwest Ethiopia; A community based cross sectional study. PLoS ONE.

[30] Haidar J., Abate G., Kogi-Makau W., Sorensen P. (2005). Risk factors for child under-nutrition with a human rights edge in rural villages of North Wollo, Ethiopia. East Afr. Med. J..

[31] Abate K.H., Belachew T. (2013). Chronic Malnutrition Among Under Five Children of Ethiopia May Not Be Economic. A Systemic Review and Meta-Analysis. Ethiop. J. Health Sci..

[32] Sapkota V.P., Gurung C.K. (2009). Prevalence and Predictors of Underweight, Stunting and Wasting in Under-Five Children. J. Nepal Health Res. Council.

[33] Reurings M., Vossenaar M., Doak C.M., Solomons N.W. (2013). Stunting rates in infants and toddlers born in metropolitan Quetzaltenango, Guatemala. Nutrition.

[34] Fenn B., Bulti A.T., Nduna T., Duffield A., Watson F. (2012). An evaluation of an operations research project to reduce childhood stunting in a food-insecure area in Ethiopia. Public Health Nutr..

[35] Medhin G., Hanlon C., Dewey M., Alem A., Tesfaye F., Worku B., Tomlinson M., Prince M. (2010). Prevalence and predictors of undernutrition among infants aged six and twelve months in Butajira, Ethiopia: The P-MaMiE Birth Cohort. BMC Public Health.

[36] Ahmadi D., Amarnani E., Sen A., Ebadi N., Corrtbaoui P., Melgar-Quinonez H. (2018). Determinants of child anthropometric indicators in Ethiopia. BMC Public Health.

[37] Humphrey J.H., Mbuya M.N.N., Ntozini R., Moulton L.H., Stoltzfus R.J., Tavengwa N.V., Mutasa K., Majo F., Mutasa B., Mangwadu G. (2019). Independent and combined effects of improved water, sanitation, and hygiene, and improved complementary feeding, on child stunting and anaemia in rural Zimbabwe: A cluster-randomised trial. Lancet Glob. Health.

[38] Cumming O., Curtis V. (2018). Implications of WASH Benefits trials for water and sanitation. Lancet Glob. Health.

[39] Morita T., Godfrey S., George C.M. (2016). Systematic review of evidence on the effectiveness of safe child feaces disposal interventions. Trop. Med. Int. Health.

[40] World Health Organisation (2011). WHO Anthropometric Macros. http://www.who.int/childgrowth/software/en/.

[41] Biswas S., Bose K. (2010). Sex differences in the effect of birth order and parents’ educational status on stunting: A study on Bengalee preschool children from eastern India. HOMO J. Comp. Hum. Biol..

[42] Edris M. (2007). Assessment of nutritional status of preschool children of Gumbrit, North West Ethiopia. Ethiop. J. Health Dev..

[43] Garcia-Granero M. (2016). Breusch-Pagan & Koenker Test Codes. http://spsstools.net/pt/syntax/442/.

